# The role of epistemic trust and epistemic disruption in vaccine hesitancy, conspiracy thinking and the capacity to identify fake news

**DOI:** 10.1371/journal.pgph.0003941

**Published:** 2024-12-04

**Authors:** Michal Tanzer, Chloe Campbell, Rob Saunders, Thomas Booker, Patrick Luyten, Peter Fonagy

**Affiliations:** 1 Research Department of Clinical, Educational and Health Psychology, University College London, London, United Kingdom; 2 Faculty of Psychology and Educational Sciences, KU Leuven, Leuven, Belgium; McGill University, CANADA

## Abstract

Epistemic trust ‐ defined as readiness to regard knowledge, communicated by another agent, as significant, relevant to the self, and generalizable to other contexts–has recently been applied to the field of developmental psychopathology as a potential risk factor for psychopathology. The work described here sought to investigate how the vulnerability engendered by disruptions in epistemic trust may not only impact psychological resilience and interpersonal processes but also aspects of more general social functioning. We undertook two studies to examine the role of epistemic trust in determining capacity to recognise fake/real news, and susceptibility to conspiracy thinking–both in general and in relation to COVID-19. Measuring three different epistemic dispositions–trusting, mistrusting and credulous–in two studies (study 1, n = 705; study 2 n = 502), we found that Credulity was associated with inability to discriminate between fake/real news. We also found that both Mistrust and Credulity mediated the relationship between exposure to childhood adversity and difficulty in distinguishing between fake/real news, although the effect sizes were small. Finally, Mistrust and Credulity were associated with general and COVID-19 related conspiracy beliefs and vaccine hesitancy. We discuss the implications of these findings for our understanding of fake news and conspiracy thinking.

## Introduction

### Epistemic trust and epistemic disruption

Epistemic trust (ET) has been defined as readiness to regard knowledge, communicated by another agent, as significant, relevant to the self, and generalizable to other contexts [for other definitions see 1,2] ). Developmental research has shown how infants are primed to respond to interpersonal signals (“ostensive cues”), which trigger openness to social learning [3–6]. Building on such experimental work, a developmental framework has been posited, arguing that disruption to the capacity for epistemic trust may drive manifestations of psychopathology and undermine healthy functioning [7,8]. It has been proposed that individuals whose interpersonal environment has left them feeling understood may be more likely to be open to social communication and present high levels of Epistemic Trust [8]). On the other hand, a maladaptive developmental environment or adverse social experiences can lead to epistemic disruption. Epistemic disruption can be expressed in high levels of Epistemic Mistrust, involving a tendency to reject or avoid any communication and/or excessive Epistemic Credulity, where information is received with insufficient discrimination, leaving the recipient vulnerable to misinformation and/or exploitation [9]. We acknowledge that our definition of *epistemic stance*, which we use to describe individual positions in relation to the reception of cultural information, differs from the broader concept of epistemology, which refers to an entire system of knowledge. Instead, we employ the term *epistemic* in line with its dictionary definition, referring to matters related to knowledge.

To explore these different constructs, we used a well validated a measure of epistemic trust in which these three hypothesised epistemic stances–Trust, Mistrust and Credulity were confirmed by exploratory and confirmatory factor analyses (the Epistemic Trust, Mistrust and Credulity Questionnaire [9] in two large samples of community adults (Study 1, n = 500; Study 2, n = 705). These three correlated yet distinct factors were shown to be associated with reported childhood experiences of trauma and mental health symptoms in adults. In particular, Mistrust and Credulity were associated with insecure attachment styles and childhood traumatic experiences, and both factors partially mediated the link between trauma and mental health symptoms [9–12]. A significant implication of these findings is that the content of interpersonal communication may be rejected, or its meaning or intent misinterpreted, as a result of epistemic disruption, associated with early adversity, thus potentially hampering social learning [13]. On the other hand, an adaptive epistemic stance–in which vigilance and trust can be appropriately mobilised in response to judgements about the quality of information or the trustworthiness of the informant–may underpin healthy functioning, which requires rapid, efficient checking and updating of social knowledge [14,15]. An open question, which this study aims to investigate, is how and if epistemic trust/disruption, as conceptualized within this developmental psychopathology framework, can help us understand individual functioning in relation to wider social communication activities such as dealing with media misinformation or judging whether to engage with conspiracy theories.

### Fake news and misinformation

There are currently high levels of concern about the breakdown in trust in legitimate sources of information and the effects of misinformation [16,17]. The COVID-19 crisis has further heightened awareness of the spread of misinformation and how conspiracy theories can affect behaviour such as adherence to public health guidance and vaccine uptake [18–20]. For example, approximately 10% of a UK representative sample showed very high levels of conspiracy thinking about COVID-19, and 50% to some degree endorsed conspiracy thinking [21]. Similarly a US-based study found that one third of their adult sample believed one or more conspiracies about COVID-19 [22].

Research into both conspiracy thinking and the capacity to recognise misinformation are fast-moving fields. Demographic features, including political affiliation, educational achievement, age, gender, ethnicity, religiosity, income, and marital status, together explain only about one third of the variance in susceptibility to COVID-19 myths [23]–hence further factors are involved. Recent attempts to identify what makes people more prone to believe or share fake news have highlighted cognitive processes in relation to information seeking [15]. For example, those with stronger analytical thinking (as measured by the Cognitive Reflection Test (CRT) [24]) appear to be less susceptible to believing fake news and better able to discriminate between real and fabricated news [15,25–27]. The relationship between performance on the CRT and fake news susceptibility has been attributed to “lazy” thinking, defined as a tendency not to consider information reflectively or override intuitive responses [25], and may indicate impulsivity in judging information [28]. Further, personality factors such as agreeableness, conscientiousness and lower levels of extraversion have been related to better truth discrimination [29], while cognitive factors such as confirmation bias [30], the effect of repetition [31] and selective exposure have been found to elevate vulnerability to fake news, especially when received via social media [26].

In relation to conspiracy beliefs, factors such as education level, income level, reduced social network, existential threats (both ongoing personal distress and perceived threats from the world around us), have been implicated in conspiracy thinking [32–34]. Douglas and colleagues also conclude that the existing literature indicates that conspiracy theories tend to attract individuals who seek meaning, certainty or exact explanations but who may also have reduced cognitive skills or are less able to find meaning in more rational sources. A recent meta-analysis found that conspiracy thinking tends to be associated with epistemic concerns, which are defined here as automatic thinking styles adopted as a response to intolerance of uncertainty [32].

It is important to note, however, that the concepts of real and fake news, vaccine hesitancy and conspiracy thinking–constructs we aim to measure in this study–are contested. These terms have been particularly problematized in the fields of global public health and medical anthropology [35,36]. In the context of COVID-19 vaccine hesitancy, Schiavo recommended: ‘Empathy, respect, cultural humility and genuine concern in discussing any doubts or fears people may have, and providing them with evidence-based information to positively shape immunization decisions should inform all our efforts’ [36, p. 4]. The mentalizing approach to psychopathology–the theoretical framework for our research–explicitly promotes a mentalizing stance that strongly echoes Schiavo’s approach: curiosity, empathy, validation, and a ‘not-knowing’ position regarding others’ mental states. Given the emerging evidence that COVID-19 mortality rates were higher in socioeconomically disadvantaged area compared to affluent ones [37]), the public health imperative to understand and challenge communicative injustice is clear.

A highly influential paper in the global public health literature on culture in health communication examined how three components in a model of health communication planning–the source of communication, the message communicated and the channel or medium–affect persuasion and are themselves affected by culture. By examining particular exercises in public health communication, the study demonstrated the value of considering cultural factors in enhancing communication effectiveness [38]. Such work is congruent with developmental studies on children’s trust in the communication of information, indicating that children are responsive to socio-cultural signals such as similarity, accent and in-group affiliation and pro-sociality, as well as epistemic cues such as past accuracy, perceived experience and sound reasoning [39]. The study undertaken here seeks to make a tentative step in integrating the literature emanating from these separate disciplines (developmental science and public health) to understand how developmental experiences, in particular exposure to adversity and./or to a social environment which fails to recognise agentive selfhood, shapes social learning. Thus the current studies seek to make a contribution to the rapidly developing area of research on factors that may generate vulnerability to believing fake news and adopting conspiracy thinking by exploring the relationship between these tendencies and individual levels of epistemic trust and epistemic disruption. Note that in relation to both conspiracy beliefs and fake news, questions of trust in the communicator of information have been explored [40,41]. For example, trust in the person or the media source sharing the information has been suggested as a potential mechanism affecting individuals’ tendency to believe and share fake news [42–44] or hold a conspiracy mindset [41]. However, no study has to our knowledge explored these relations from the recipient perceptive, i.e., how individual epistemic stance is associated with responses to fake news and conspiracy beliefs, and whether exposure to childhood adversity may be implicated in the nature of the individual’s epistemic stance. To explore these relationships, we undertook two separate studies on epistemic stance, exposure to childhood adversity, accuracy in detecting fake news, conspiracy thinking and vaccine hesitancy. Both studies’ hypotheses, sample size and design were preregistered (https://osf.io/ef695; https://osf.io/s72m3).

## Study 1

The hypotheses developed for this study are predicated on the idea that exposure to unhelpful informants (such as via traumatic childhood experiences) will affect the development of an individual’s epistemic stance, affecting later functioning when it comes to reasoning about the trustworthiness of information.

Our detailed hypotheses were as follows:

(H1) Following Imhoff and colleagues’ work on the relationship between conspiracy mindset and judgements about epistemic credibility [41], we hypothesized that epistemic disruption (i.e., high Credulity/Mistrust) would be positively associated with higher conspiracy mentality.
(H2) Consistent with previous findings on the relationship between reduced analytical thinking (measured by the CRT) and failure to detect fake news [25], we hypothesised that epistemic disruption would be associated with reduced analytical thinking.
(H3) We hypothesised that individuals with high epistemic disruption would be more likely to struggle to distinguish real from fake news after controlling for their analytical thinking.
(H4) Based on the proposed role of exposure to an unreliable social environment in which informants cannot be assumed to be helpful, we predicted Trust, Mistrust and Credulity to mediate the expected associations between levels of childhood trauma and scores on the fake news task. Specifically, that higher levels of childhood trauma would be associated with reduced ability to distinguish real from fake news, and that this association would be mediated by epistemic stance.
(H5) Finally, as the study was conducted during the COVID-19 pandemic we also sought to test the relationship between epistemic stance and conspiracy beliefs in relation to the pandemic, and anticipated that people with epistemic disruption would score higher on COVID-19 related conspiracy beliefs.

### Methods

#### Ethics statement

The study was undertaken in accordance with the ethical standards of the UCL Research Ethics Committee, from whom ethical approval was obtained (14285/002).

#### Participants and procedures

A total of 705 participants took part, using the on-line survey platform Prolific (https://www.prolific.co), which allowed us to recruit a representative sample that approximately matches the United Kingdom population distribution in terms of age, sex and ethnicity. Recruitment and data collection took place in June 2020. Sample size was calculated based on a prior related medium effect size [25] using GPower (G-power 3.1) with 95% power, .05 alpha and added contingency of 20–25%. Participants were aged 18 years or older, currently living in the UK, and proficient in written and spoken English (S1 Table). Participants received financial compensation (at a rate of £7.50/hour). Questionnaires were designed in Qualtrics (Qualtrics, Provo, UT). Participants were first asked to complete the demographic questions, followed by a battery of questionnaires presented in randomized order including the fake news task. For ethical reasons, at the end of the study, we posted links to fact-checking websites (such as fullfact.org).

#### Instruments

*Epistemic stance*. To evaluate participants’ openness to the communication of knowledge we used the epistemic Trust, Mistrust and Credulity questionnaire (ETMCQ [9]). The ETMCQ is a 15-item questionnaire and responses are rated across a 7-point Likert scale ranging from “strongly disagree” (= 1) to “strongly agree” (= 7) and neither agree nor disagree in the center (= 4). A Trust item is “I find information easier to trust and absorb when it comes from someone who knows me well”. An example of a Mistrust item is “If you put too much faith in what people tell you, you are likely to get hurt”. A Credulity item is “When I speak to different people, I find myself easily persuaded even if it is not what I believed before”. Cronbach’s α were .70, .65 and .81, respectively.

*Conspiracy beliefs*. To evaluate participants’ beliefs in general conspiracy theories we used the Conspiracy Mentality Questionnaire (CMQ [45]). The CMQ is A 12-item self-report scale using a 7-point Likert scale to assesses non-content specific tendency towards conspiratorial thinking (e.g., “There are secret organizations that have a great influence on political decisions”). In the present study, Cronbach’s α was .83.

*Cognitive reflection*. To assess cognitive reflection we used a 7-item questionnaire that measures reflective reasoning and differences in intuitive-analytic cognitive styles (CRT [24,46]). Correct responses reflect more analytical-reflective thinking while incorrect intuitive response can indicate an overall failure to engage in reflective reasoning processes [25]. Due to a technical error (presenting the participants the wrong unit for the answer) we had to omit one question and CRT scores were calculated based on six items.

*Fake/Real news task*. Participants were presented with 20 politically neutral news headlines. Headlines were chosen based on Pennycook and Rand’s study, with additional headlines adapted to the UK sample. The accuracy rate of the headlines was tested and validated using independent colleagues. Each headline was presented as a picture accompanied by short text and a reference to a source in a social media format. Participants were instructed to rate to what extent they think these headlines are accurate using a 4-point Likert scale (from “Not at all accurate” to “Very accurate”) and whether they would be willing to share this news item on social media using a 3 options (“Yes”, “No” and “Maybe”). This task and the scales were adapted from Pennycook and Rand [25]. All headlines were checked and taken from snopes.com or FullFact.org. Real news headlines were also fact-checked. We calculated the score of “Truth-discrimination” by subtracting the standardized score for fake news (false alarms) from the standardized score for real news (hits) [25]. A positive score on this measure indicates a capacity to distinguish real from fake news (see S1 Fig for the stimuli that were used). Results showed that Fake news headlines were perceived as less accurate than the real headlines (*M*_fake_ = 1.75, *SD* = .37; *M*_real_ = 2.51, *SD* = .37, *p* < .001, 95% CI [-.78,-72]). Among individuals who reported that they sometimes share news over social media (n = 535), willingness to share fake news was lower than real news (*M*_fake_ = 0.21, *SD* = .44; *M*_real_ = 0.56, *SD* = .47; *p* < .001, 95% CI [32,38]).

*Childhood trauma*. The experience of trauma in childhood was measured using the Childhood Trauma Questionnaire, a 28-item self-report questionnaire validated for clinical and non-clinical populations [47]. Individuals are asked to indicate on a 5-point Likert scale whether and how often they experienced emotional, physical or sexual abuse and emotional or physical neglect in their childhood. Cronbach’s α were .90, .71, .84, .89 and .95, respectively. Twenty-two participants (2%) had one missing item which was replaced by the subscale mean response items. Four (0.1%) participants had two missing items which were replaced by the subscale mean response items. One participant skipped more than four items and as such was excluded from analysis of this measure (S1 Text for percentage of participants reporting on childhood traumatic experiences within our sample).

*Coronavirus conspiracy explanations*. Participants’ conspiracy beliefs in relation to the COVID-19 crisis were measured using a newly developed scale [21] in which participants are asked to rate the extent to which they agree with 48 COVID-19 conspiracy statements (e.g. “I’m skeptical about the official explanation about the cause of the virus”). The questionnaire authors group the items into six subscales: skepticism about the government’s response, general conspiracy views on the cause of the virus, general conspiracy views about the spread of the virus, general conspiracy views about the reasons for lockdown, specific conspiracy beliefs, and level of agreement with official explanations.

#### Data analysis

We first ran descriptive statistics and as the ETMCQ scores violated assumptions of normality, we used nonparametric statistical tests. Spearman’s rho correlation analyses were performed to assess the associations between the ETMCQ subscales, CMQ, demographic information (i.e., age, level of education and annual income) and CRT. In addition, we examined the relationships between demographic factors and scores on the fake news task.

To test our hypothesis on the association between the ETMCQ subscales, Truth-discrimination and perception of fake/real news headlines, we conducted three regression models with ETMCQ and conspiracy beliefs as independent variables and sex, age, education, income and CRT as covariates. Distribution residuals were checked following model construction to check for violation of normality residuals and multicollinearity was tested. Results were corrected for multiple comparisons [48].

The role of the three ETMCQ subscales as mediators between childhood trauma (measured by the CTQ total score) and fake/real discrimination was explored using mediation analysis with the indirect effect of childhood traumatic experiences through each of the factors estimated separately. Age, sex, income, CRT and level of education were included as covariates. Bias-corrected bootstrapped confidence intervals (BC-95%Cis) for the indirect effects were estimated (5000 bootstrap replications).

To explore the hypotheses relating to the COVID-19 scale, correlational analyses of ETMCQ with general and COVID-19 specific conspiracies beliefs, whilst controlling for age, sex, annual income, CRT and level of education, were conducted and corrected for multiple comparisons [48].

### Results

#### Epistemic stance, conspiracy beliefs and analytical thinking

Our first hypothesis (H1) regarding the association between epistemic disruption (i.e., Mistrust and Credulity) and conspiracy mentality was confirmed: Mistrust and Credulity were positively correlated with conspiracy beliefs (*r*_(705)_ = .30, *p* < .001, 95%CI[.23,37]; *r*_(705)_ = .23, *p* < .001, 95%CI[.17,30] respectively) (S2 and S3 Tables for summaries of Spearman intercorrelations). The second hypothesis (H2) regarding the association between epistemic stance (i.e., Trust, Mistrust and Credulity) and analytical thinking was partly confirmed, with individuals scoring high on Credulity showing reduced cognitive reflection (*r*_(705)_ = -.12, *p* = .001; 95%CI[-.05;-.17]), although this correlation was small. Moreover, there were no significant correlations between Trust and Mistrust and analytical thinking as measured by the CRT score ((*r*_(705)_ = -.06, *p* = .13; 95%CI[-.13;0.02]); (*r*_(705)_ = -.00, *p* = .92; 95%CI[-.01;.01]), respectively).

#### Epistemic stance and fake/real news task scores

As predicted (H3), when Truth-discrimination (i.e., the capacity to distinguish real from fake news) served as the dependent variable, a main effect for analytical thinking, as measured by the CRT score, emerged (Table 1), suggesting that individuals with a higher score on the CRT were better at distinguishing real from fake news. Similarly, and consistently with expectations, Credulity was associated with lower Truth-discrimination, suggesting that individuals with a higher score on the Credulity subscale were less able to distinguish real from fake news. To further investigate these effects, regression analysis was performed with perception of fake news as accurate as the dependent variable (e.g., False alarms) (Table 1). Credulity again emerged as a significant main effect, indicating that Credulity was associated with incorrectly perceiving fake news as real. Both conspiracy mentality thinking and CRT emerged as significant main effects, suggesting that individuals with higher scores on these measures were more likely to perceive fake news as real. Finally, when the hit rate of recognising real news as accurate was the dependent measure, Trust was positively associated with accurate recognition (b = .09, SE = .05, *p* = .04, 95%CI[.00;.04]), but this did not survive FDR corrections. Against expectations, we did not find any significant effect for Mistrust (Table 1).

**Table 1:** A. Correlations between ETMCQ subscales, conspiracy beliefs, score on the CRT and dependent variables [Truth-discrimination, Fake accuracy (False alarm) and Real news accuracy (Hit)] B. Linear multiple regression of ETMCQ subscales on the fake/real measures (n = 475), controlling for sex, education, income, CRT and age, FDR corrected.

A.					Trust			Mistrust		Credulity		CMQ	CRT	
Truth-discrimination					.05			-.06		-.15^**^		-.15^**^	.20^**^	
Fake accuracy (False alarm)					.02			.06		.13^**^		.19^**^	-.21^**^	
Real news accuracy (Hit)					.08^*^			-.00		-.05		.01	.04	
B. Dependent variable ‐ Truth-discrimination; *R*^*2*^ = 0.08														
Independent Variables		*b*		*SE*			*β*		*t*		*P*	Collinearity Tolerance		VIF
Trust		.07		.06			.05		1.26		.25	.86		1.16
Mistrust		-.02		.06			-.02		-.41		.68	.76		1.32
Credulity		**-.15**		**.05**			**-.14**		**-3.30**		**.002**	**.81**		**1.23**
CMQ		-.09		.05			-.08		-1.96		.09	.85		1.17
CRT		**.67**		**.15**			**.17**		**4.36**		**.0001**	**.90**		**1.10**
Dependent variable–Fake accuracy (False alarm), *R*^*2*^ = 0.09														
Trust	0.00		.01			.02			.43		.75	.86		1.15
Mistrust	0.00		.01			.01			.32		.75	.75		1.32
Credulity	**0.01**		**.01**			**.10**			**2.33**		**.03**	**.81**		**1.23**
CMQ	**0.02**		**.01**			**.14**			**3.53**		**.001**	**.85**		**1.18**
CRT	**-0.09**		**.02**			**-.18**			**-4.70**		**.0001**	**.90**		**1.10**
Dependent variable–Real news accuracy (Hit); *R*^*2*^ = 0.04														
Trust	0.2		.01			.08			2.03		.15	.86		1.15
Mistrust	-0.00		.01			-.01			-0.21		.85	.76		1.32
Credulity	-0.01		.01			-.08			-1.87		.15	.81		1.24
CMQ	0.01		.01			.04			1.03		.46	.85		1.17
CRT	0.02		.02			.04			0.86		.48	.90		1.11

Note: CRT = cognitive reflection test; CMQ = conspiracy mentality questionnaire; ETMCQ = epistemic trust, mistrust and credulity questionnaire; FDR = false discovery rate.

#### The mediating role of epistemic stance on the relationship between childhood trauma and fake/real task scores

**Fig 1:**
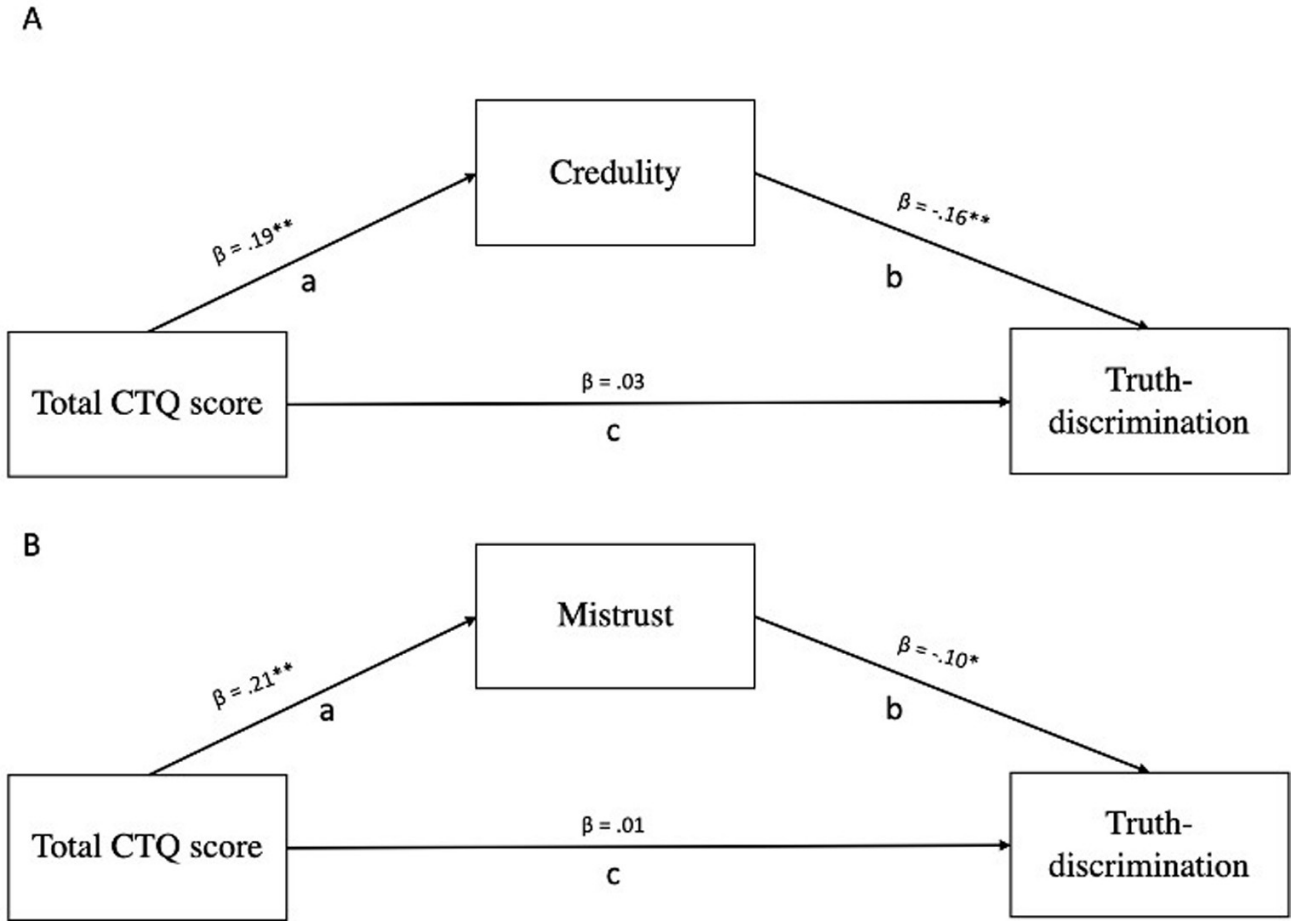
Schematic model of the mediating role of Credulity (A) and Mistrust (B) on the relationship between childhood trauma as measured by the Childhood Trauma Questionnaire (CTQ), and Truth-discrimination. Controlling for age, gender, education, Cognitive Reflections Task (CRT) and income. Study 1 n = 663 *p < .01 **p < .001.

To test the mediating effect of epistemic disruption on the relationship between exposure to childhood trauma and Truth-discrimination (as measured by the Fake/Real task) (H4), we first tested the relationships between retrospective reports on exposure to childhood trauma (as measured by the total score on the childhood trauma questionnaire (CTQ)) and Truth discrimination. Despite not finding a direct effect (see below), nor a correlation between these measures (*r*_(704)_ = -.04, *p* = .31; 95%CI[-.11;0.04]), we did find a mediating effect. Both Credulity and Mistrust mediated the relationship between the total score on the CTQ and Truth-discrimination (b = -0.003, SE = 0.001, 95%[-0.004; -0.001];b = -0.001, SE = 0.001, 95%[-0.004; -0.002] see Fig 1A and 1B). Specifically, score on the CTQ was a significant predictor of Credulity (path a in Fig 1A; *b* = .01, *SE* = .003, *p* < .001, 95%CI[0.01;0.02]), and Credulity was a significant predictor of Truth-discrimination (path b in Fig 1A; *b* = -0.18, *SE* = .04, *p* < .001, 95%CI[-.27;-.09]). Similarly, score on the CTQ was a significant predictor of Mistrust (path a in Fig 1B; *b* = .01, *SE* = .00, *p* < .001, 95%CI[0.01;0.02]), and Mistrust was a significant predictor of Truth-discrimination (path b in Fig 1B; *b* = -0.14, *SE* = .01, *p* = .01, 95%CI[-.25;-.03]). For both models, the direct effect between CTQ score and Truth-discrimination was not significant (path c in Fig 1A and 1B; b = 0.002, SE = .003, p = 0.50 95%[-0.004;0.01]; b = 0.001, SE = .003, p = 0.71 95%[-0.00;0.01]), which indicates an indirect only mediation [49]. We did not find any mediation effect with Trust (*b* = -0.05, 95%CI[-.00;-.00]).

#### Epistemic stance and conspiracy beliefs regarding COVID-19

Mistrust and Credulity were positively associated with conspiracy beliefs regarding COVID-19 (H5; Table 2). Trust was positively associated with agreement with official explanations about the virus (*r*_(664)_ = .09, *p* = .01, 95%CI[0.01;0.18]) and negatively with lockdown conspiracies (*r*_(664)_ = -.09, *p* = .02, 95%CI[-0.01;-0.17]), but these effects did not survive FDR corrections. Scores on the Conspiracy Mentality Questionnaire (CMQ) were positively associated with COVID-19 conspiracy beliefs and negatively with agreement with the official explanations (Table 2) (percentages of our sample reporting on believing in the specific conspiracies are reported in S2 Text).

**Table 2:** Spearman correlations between ETMCQ subscales, conspiracy beliefs, and conspiracy beliefs in relation to COVID-19 controlling for CRT, sex, age, annual income and level of education (FDR corrected).

	Official explanations	Skepticism Authorities	Conspiracies on			
			Cause of the virus	Lock-down	Specific COVID-19	Spread of the virus
1. Trust	.10	-.03	.00	-.09	-.06	-.05
2. Mistrust	.-.05	.16^***^	.09^*^	.14^***^	.19^***^	.16^***^
3. Credulity	-.04	.15^***^	.11^***^	.14^***^	.17^***^	.16^***^
4. CMQ	-.23^**^	.49^***^	.42^***^	.39^***^	.52^***^	.51^***^
5. Truth dis	.16^*^	-.24^***^	-.25^**^	-.18^***^	-.18^***^	-.23^***^

Note: CRT = cognitive reflection test; CMQ = conspiracy mentality questionnaire; ETMCQ = epistemic trust, mistrust and credulity questionnaire; FDR = false discovery rate.

^*^p < .05.

^**^p < .01.

^***^p < .001.

### Discussion

With the aim of further understanding what makes some individuals more prone to believe in fake news and adopt conspiracy thinking, we tested the relations between these tendencies and individual levels of epistemic trust and epistemic disruption. Epistemic disruption (both Mistrust and Credulity) was found to correlate with belief in conspiracy theories; individuals with high Credulity were poorer at discriminating between fake and real news and more likely to perceive fake news as real and affirm false news in relation to COVID-19. Higher Trust was associated with correctly identifying real news headlines but these effects did not survive statistical correction. Findings on the associations between epistemic stance and believing in fake news and conspiracy theories are consistent with previous findings that support the role of psychological factors in increasing vulnerability to both phenomena [15,33]. Our hypothesis regarding the role epistemic disruption as a mediator in the relationship between retrospective reports of childhood trauma and the ability to discriminate between fake from real news was partially confirmed in that the effect size we found was small. In order to further test these relationships and given that this study was the first to test these relations, we set out to replicate and extend these findings (see Study 2).

## Study 2

Study 2 sought to extend the findings of Study 1. Firstly, to explore the mediating role of epistemic disruption on fake news discrimination in individuals who have experienced trauma, we used a different scale to measure exposure to traumatic experiences in childhood, the Maltreatment Abuse and Exposure Scale (MAES). This scale was chosen on the grounds that it captures severity as well as exposure, and shows greater correlation with psychopathology symptoms than the CTQ [50], which was thought might result in larger effect sizes. Secondly, the ongoing COVID-19 pandemic and the introduction of a vaccine programme for COVID-19 allowed us to investigate the relationship between epistemic stance and specific COVID-19 conspiracies.

Our Study 2 hypotheses were the following:

(2H1) Study 1 findings on the relationships between epistemic stance (i.e., levels of Trust, Mistrust and Credulity) and scores on the fake news task would be replicated. We also hypothesised that our findings on the mediating effect of epistemic disruption (i.e., Mistrust and Credulity) on the relationship between exposure to traumatic experiences in childhood and truth discrimination would be replicated.

(2H2) We hypothesised that epistemic disruption would be associated with vaccine hesitancy, both generally and in relation to COVID-19, and that Trust would not be associated with vaccine hesitancy.

### Methods

#### Ethics statement

The study was undertaken in accordance with the ethical standards of the UCL Research Ethics Committee, from whom ethical approval was obtained (14285/002).

#### Participants and procedures

A total of 502 participants from a representative UK sample took part, using the procedure described in Study 1 (see S1 Table for demographic characteristics and preregistration for sample size). As in study 1, at the end of the study, we posted links to fact-checking websites (such as fullfact.org) and to official information and guidance on vaccines from the UK National Health Service.

#### Instruments

*Epistemic stance*. We used the ETMCQ, as described in Study 1. Cronbach’s α for Trust, Mistrust and Credulity were .73, .69 and .70, respectively.

*Vaccination attitude examination*. Participants’ attitudes towards vaccination were evaluated using the Vaccination Attitude Examination (VAX [51]). The VAX is A 12-item self-report scale using a 6-point Likert scale to assesses general beliefs towards vaccinations and has been shown to be associated with vaccination behaviours and intentions [51]. In the present study, Cronbach’s α was .71.

*COVID-19 vaccination*. Willingness to vaccinate against COVID-19 was measured using one item (“If a COVID-19 vaccine were made available to me, I would definitely get it?”). If participants had already received the vaccination, they were requested to report how they felt before they were offered it. Response option was ranged on a 6-point Likert scale. Confidence in the safety and efficacy of the COVID-19 programme was based on one item ("I have confidence in the safety and efficacy of the COVID-19 vaccination programme"), using the same scale.

*Fake/Real news task*. See Study 1.

*Childhood Trauma*. To establish individuals’ exposure to trauma we used the Maltreatment Abuse and Exposure Scale (MAES), a 52-item questionnaire that assesses the severity of exposure to ten types of maltreatments. Responses were scored and validated using the MAES protocol, resulting in the exclusion of 31 participants (6%).

#### Data analysis

Data analysis was identical to study 1, with the exception of the use of the additional instruments reported above.

### Results

#### Epistemic stance and fake/real news task scores

Similarly to Study 1 findings, and confirming our hypothesis (2H1) regarding the association between epistemic stance (i.e., Trust, Mistrust and Credulity) and Truth-discrimination, we found that when Truth-discrimination served as the dependent variable, Credulity emerged as a negative significant main effect (see Table 3). Specifically, individuals with a higher score on the Credulity subscale were less able to distinguish real from fake news. When perception of fake news as accurate served was the dependent variable (i.e., False alarm), Credulity again emerged as a significant main effect, indicating that those with high Credulity were more prone to perceive fake news as real. Similarly, a positive main effect for Mistrust emerged, indicating that those with high Mistrust were more prone to perceive fake news as real.

**Table 3:** A. Spearman correlations between ETMCQ subscales and dependent variables [Truth-discrimination, Fake accuracy (False alarm) and Real news accuracy (Hit)] B. Linear multiple regression of ETMCQ subscales on the fake/real measures (n = 472), controlling for sex, education, income and age, FDR corrected.

A.					Trust			Mistrust	Credulity				
Truth-discrimination					.07			-.11*	-.14^**^				
Fake accuracy (False alarm)					-.02			.14^**^	.17^**^				
Real news accuracy (Hit)					.09^*^			.01	.03				
B. Dependent variable ‐ Truth-discrimination; R^2^ = 0.07													
Independent Variables		*b*		*SE*			*β*		*t*		*p*	Collinearity Tolerance	VIF
Trust		.04		.07			.03		0.63		.52	.79	1.27
Mistrust		-.13		.06			-.10		-1.77		.08	.70	1.42
Credulity		**-.13**		**.05**			**-.12**		**-2.41**		**.02**	**.83**	**1.20**
Dependent variable–Fake accuracy (False alarm); R^2^ = 0.07													
Trust	.007		.01			.04			.73		.46	.78	1.27
Mistrust	**.02**		**.01**			**.12**			**2.21**		**.03**	**.70**	**1.42**
Credulity	**0.02**		**.01**			**.15**			**3.13**		**.002**	**.83**	**1.20**
Dependent variable–Real news accuracy (Hit); R^2^ = 0.03													
Trust	0.17		.01			.08			1.50		.39	.79	1.27
Mistrust	0.00		.01			.00			0.04		.98	.70	1.42
Credulity	0.00		.01			.01			.18		.98	.83	1.20

Note: ETMCQ = epistemic trust, mistrust and credulity questionnaire; FDR = false discovery rate.

#### The mediating role of epistemic stance on the relationship between childhood trauma and fake/real task scores

**Fig 2:**
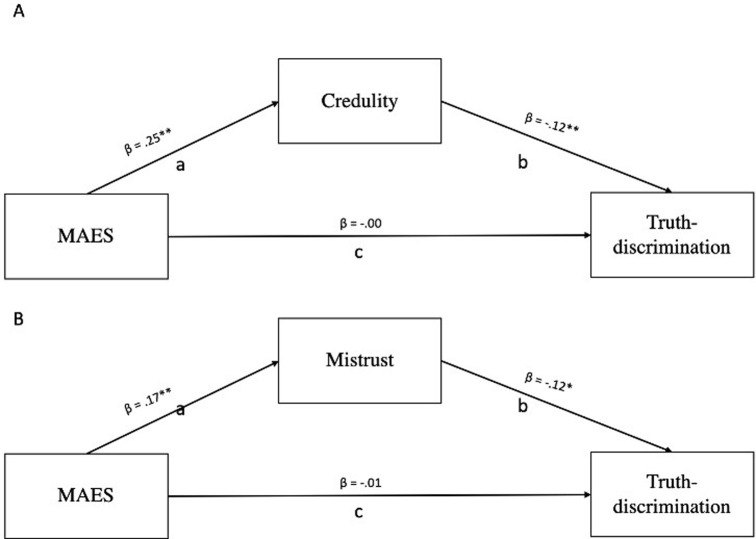
Schematic model of the mediating role of Credulity (A) and Mistrust (B) on the relationship between childhood trauma as measured by the Maltreatment Abuse and Exposure Scale (MAES), and Truth-discrimination. Controlling for age, gender, education and income. Study 2 n = 443 *p < .01, **p < .001.

In relation to childhood trauma (2H1), similarly to Study 1, Credulity and Mistrust mediated the relationship between retrospective reports on exposure to childhood trauma as measured by the MAES and Truth-discrimination (Credulity: b = -0.002, SE = 0.001, 95%[-0.005; -0.003]; Mistrust: b = -.002, SE = .00, 95%CI[-.003; -.0002], see Fig 2). As in Study 1, and despite the use of a different scale to measure childhood trauma, the effect sizes were small, and again we did not find a direct link between score on the MAES and Truth-discrimination (Credulity: b = -0.004, SE = .004, p = 0.92 95%[-0.008;0.01]; Mistrust: b = -0.00, SE = .003, p = 0.70 95%[-0.01;0.01]; see Fig 2).

#### Epistemic stance, conspiracy theories and vaccine hesitancy

Our hypothesis (2H2) on the effects of epistemic stance on attitudes towards vaccination was confirmed. Both Credulity and Mistrust were positively associated with vaccine hesitancy (*r*_(469)_ = .22, *p* < .001, *95%CI*[.13;.31] and *r*_(469)_ = .21, *p* < .001; *95%CI*[.12;.30] respectively (whilst controlling for age, sex, education level and income)). Similarly to study 1, Trust was not associated with vaccine hesitancy (*r*_(469)_ = -0.05, *p* = .27, *95%CI*[-.14;.03]). In addition, Credulity and Mistrust were both negatively correlated with willingness to have the COVID-19 vaccine (*r*_(469)_ = -.16, *p* = .001; *95%CI*[-.07;-.25] and *r* = -.13, *p* = 006, 95%CI[-.04;-.21], respectively), but only Credulity was negatively associated with confidence in the safety and efficacy of the COVID-19 vaccination programme (*r*_(469)_ = .14, *p* = .001; *95%CI*[-.05;-.24]; *r* = -0.08, *p* = 0.09; 95%CI[.02;.18], respectively). Again, Trust was not associated with these COVID vaccine hesitancy measures (*r*_(469)_ = .09, *p* = .14; 95%CI[.00;.18]; *r* = 0.04, *p* = 0.37; 95%CI[-.05;.13], respectively). (S3 Text for overall response distribution within this sample.)

#### The mediating role of epistemic stance on the relationship between childhood trauma and vaccine hesitancy

Given the above mentioned effects of epistemic disruption in mediating the relationship between exposure to trauma and truth-discrimination, a post-hoc exploration of the association between exposure to trauma, as measured by the total score on the MAES, and misinformation, as measured by vaccine hesitancy, was conducted. Both Credulity and Mistrust were found to mediate this relationship (indirect effects: Credulity: *b* = -0.002, *SE* = .00, *p* = .02, *95%CI*[.00;.004] and Mistrust: *b* = -0.002, *SE* = .00, 95%CI[.00;003]), with small effect sizes. Again, the direct effect between MAES and vaccine hesitancy was not significant (*b* = 0.005, *SE* = .002, *p* = .10, 95%CI[-.00;.01]; *b* = 0.005, *SE* = .002, *p* = .07, 95%CI[-.00;.01], respectively).

### Discussion

Findings indicate that individuals with high Credulity are less able to discriminate between fake and real news and more prone to perceiving fake news as real, replicating our findings in study 1. In addition, we again found that while there was no direct association between childhood adversity and Truth-discrimination, an indirect effect was present via the mediation of Credulity and Mistrust. However, despite our effort to shed light on causality by looking at retrospectively reported early adversity as an antecedent of epistemic disruption, the effect sizes were again small. Our hypothesis that individuals with epistemic disruption would show greater hesitancy towards vaccination was confirmed. Again, there was no direct association between childhood adversity and vaccine hesitancy but there was an indirect effect via the mediation of Credulity and Mistrust.

## General discussion

The two studies described here investigated the relationship between epistemic stance (i.e., Trust, Mistrust and Credulity) and the ability to recognise fake news and the tendency for conspiracy thinking. Using a UK representative sample, we investigated these relationships in five areas: fake news headlines, conspiracy thinking in general, conspiracy thinking about COVID-19, vaccine hesitancy in general, and COVID-19 vaccination hesitancy. We also explored the potential mediating role of the different epistemic stances in the relationship between childhood trauma and fake news discrimination.

Our findings confirmed that a higher score on the Credulity subscale was consistently associated with a reduced capacity to identify fake news and increased conspiracy thinking. We have previously suggested that the Credulity subscale captures a lack of capacity to judge accurately the quality of social communication and renders the individual more at risk of being misled or manipulated [9]. The findings in the current studies indicate that the vulnerability engendered by Credulity may not only impact psychological resilience and interpersonal processes [8] but also aspects of more general social functioning.

Although previous findings indicate an association between trauma and heightened Mistrust and Credulity [9], and the current finding showed that Credulity is associated with reduced ability to discern fake information, we did not find a significant direct effect between childhood trauma and Truth-discrimination or vaccine hesitancy. However, there was a significant small indirect effect via both Credulity and Mistrust. These findings may indicate that a disruption in the capacity to trust in the communication of information is one of the mechanisms by which childhood experiences may affect later social cognitive processes involved in the assessment of the trustworthiness of information. Given the small effect size and the correlational nature of this study we can only speculate on the direction of this effect, however it is consistent with Shafto and colleagues’ emphasis on reasoning about informants’ intent as a precursor of epistemic trust [52,53]. Further, a cross-sectional study investigating exposure to childhood adversity (again by self-report and retrospectively, using a different measure) in a large UK study (n = 2,285) found that childhood adversity counts were independently related to low trust in NHS COVID-19 information, feeling unfairly restricted by government and ending mandatory face coverings [19].

In relation to our null finding on Trust not being associated with better recognition of fake news or lower conspiracy thinking, this may be consistent with previous longitudinal research suggesting that media trust does not protect individuals against misinformation (44). Caution is required in the interpretation of such a null finding, but it could theoretically be understood in terms of an evolutionary mismatch: our social cognitive capacities were adapted to small social groups where communication took place face to face with known others. We are possibly not well-equipped to recognise when, in the context of modern media stimuli, it is advisable to close the channel of ET.

The COVID-19 pandemic allowed us to measure the effect of epistemic stance in relation to public health messages and vaccine hesitancy. Individuals with higher Credulity and Mistrust were more likely to believe COVID-19 conspiracy theories, showed greater skepticism toward official accounts and were less willing to receive the COVID-19 vaccine or to believe in the safety of the vaccination programme. Again, individuals with high Trust were not immune to conspiracy beliefs but we report a trend showing more alignment with official explanations of the origins and modes of transmission of COVID-19. This point is consistent with previous findings [19] that Trust does not act as a resilience factor for psychopathology, but rather Mistrust and Credulity constitute vulnerability factors. Trust may increase the likelihood of accepting an ‘official version’ of events [54] but does not protect us from being influenced by fake news or conspiracy theories. Thus effective interventions in public health may need to directly tackle and attempt to reverse Mistrust and Credulity. The role of Credulity indicates the readiness of a significant proportion of individuals to believe narratives without requiring evidence: it is unlikely that presenting contrary evidence alone is likely to reverse beliefs. In addition, individual differences in the ability to recognize real or fake news must be understood in the broader context of cultural factors. The literature in medical anthropology has explored the concept of communicative justice in relation to public health, Briggs’s work being particularly influential. As Briggs argues: ‘We need to bury the Lockean legacy, which decrees that communication requires diagnoses of miscommunication and interventions whose ethical value is assured in advance by the claim that they are designed to fix things. Power hierarchies are reinscribed by these instrumentalist logics, and efforts to achieve “efficacy” that do not value justice can exacerbate inequities’ [35, p.270]. Although arising from a different discipline, developmental psychopathology, we suggest that the current research contributes to communicative justice in public health by positioning individuals’ epistemic stance as an adaptation to environmental cues. Earlier iterations of our work focused on the caregiver-infant relationship in shaping the developmental environment [55]. More recently, we have emphasized the role of the broader social environment in determining an individual’s sense of agency and selfhood, with implications for their epistemic stance [8,56,57]. We have argued that disruptions in epistemic trust result from failures in communication by the communicating authority [58]. For an individual’s sense of purposeful connection to their broader social community to develop–characteriszed by an openness to social learning underpinned by epistemic trust–the individual must feel recognized as an agent through experiences of being accurately mentalized within that social system [8, p.4]. Individuals who have not experienced such recognition, and who have not been exposed to ostensive cues that promote social learning are likely to seek knowledge from alternative sources or, as an adaptative response, resist new learning altogether.

This study has some limitations. Firstly, both studies are cross-sectional and so we cannot infer causality or the involvement of a common cause in a third variable; further longitudinal research is required. We cannot rule out other factors that might affect the capacity to recognise fake news–for example, we did not assess general interpersonal trust [59], which would generate discriminant validity of the ETMCQ factors. Secondly, a possible limitation arises from the modest correlations we found between COVID-19 conspiracy beliefs and ETMCQ, suggesting that other factors may be at play. Thirdly, the studies were mainly self-report based, using online questionnaires; an experimental approach would allow us to manipulate epistemic stances and compare them with response to information/misinformation and conspiracy beliefs. Finally, this study was only UK-based; future studies should explore these questions internationally. In the current climate of concern about the loss of public trust in official discourse, especially in relation to COVID-19, the findings of this study could potentially inform perspectives on the role of trust in the communication of information.

## Supporting information

S1 Fig (TIFF)

S1 Table (XLSX)

S2 Table (XLSX)

S3 Table (XLSX)

S1 Text (DOCX)

S2 Text (DOCX)

S3 Text (DOCX)
