# Supplementary figures and images for: The role of epistemic trust and epistemic disruption in vaccine hesitancy, conspiracy thinking and the capacity to identify fake news
Source: PLOS Glob Public Health. 2024 Dec 4;4(12):e0003941. doi: 10.1371/journal.pgph.0003941 (PMC11616851; doi:10.1371/journal.pgph.0003941)

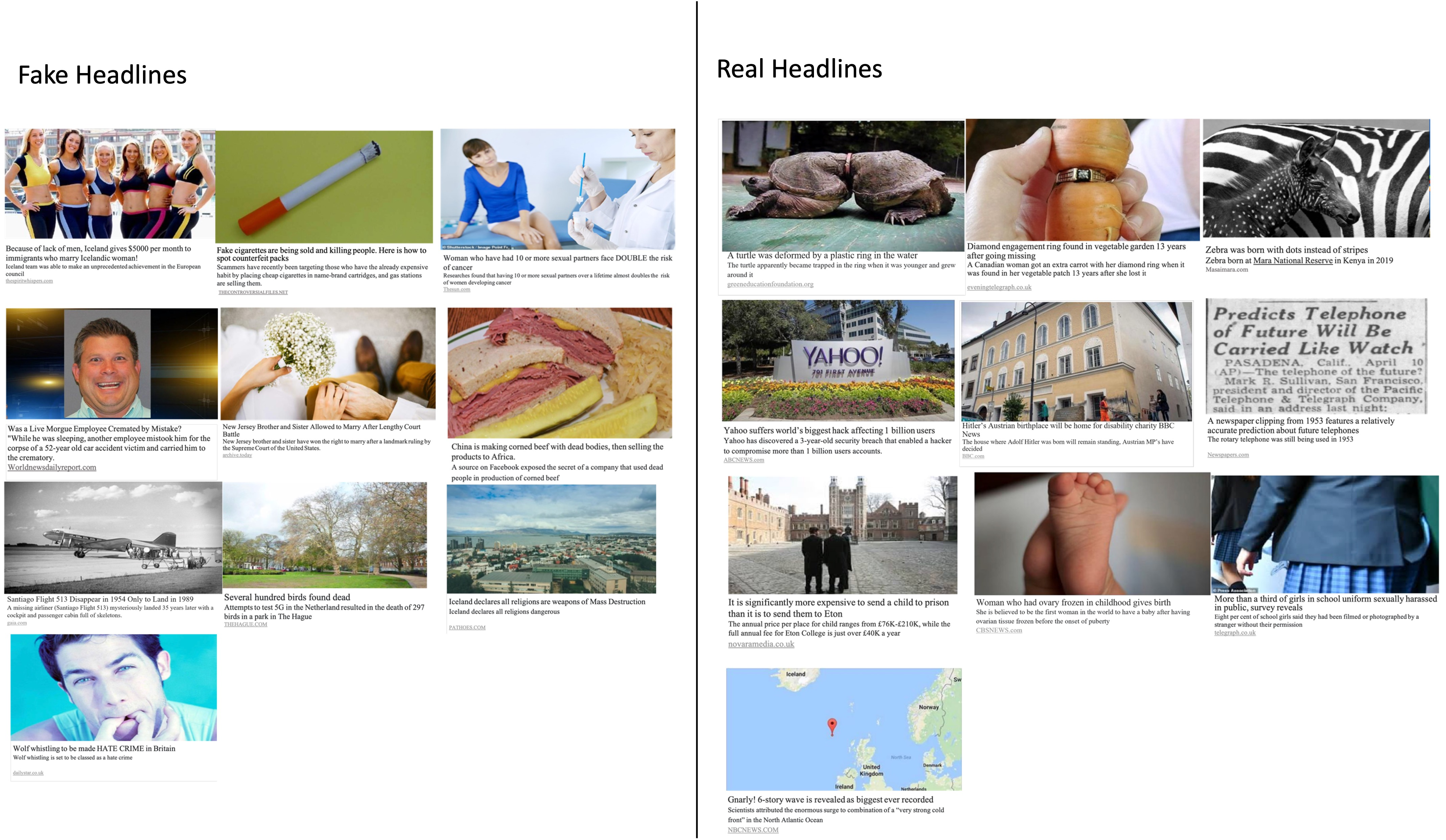

Supplement: S1 Fig — (TIFF) [file pgph.0003941.s001.tiff]
