# Supplementary material for: The role of epistemic trust and epistemic disruption in vaccine hesitancy, conspiracy thinking and the capacity to identify fake news
Source: PLOS Glob Public Health. 2024 Dec 4;4(12):e0003941. doi: 10.1371/journal.pgph.0003941 (PMC11616851; doi:10.1371/journal.pgph.0003941)
Supplement: S1 Text — (DOCX) [file pgph.0003941.s005.docx]

**Percentage of participants reporting on childhood adversity within our sample:**

**Study 1:** Within our sample, 27% of participants in the study reported moderate to severe emotional neglect (score > 15) (Bernstein & Fink, 1998), 12% of the study sample reported moderate to severe physical neglect (score >10), 11% reported moderate to severe physical abuse (score > 10), 12% of participants reported moderate to severe emotional abuse (score >13), and 10% reported moderate to severe sexual abuse (score > 8).

**Study 2:** Within our sample, 31% of participants reported on zero exposure to types of maltreatment, 17% of the study sample reported on exposure to one type of maltreatment, , 15% of participants reported on exposure to two types, 10% of participants reported on three types, 7% reported on four types, 6% on five types and 7% of participants reported on exposure to more than 7 types of maltreatment.
