# Supplementary material for: The role of epistemic trust and epistemic disruption in vaccine hesitancy, conspiracy thinking and the capacity to identify fake news
Source: PLOS Glob Public Health. 2024 Dec 4;4(12):e0003941. doi: 10.1371/journal.pgph.0003941 (PMC11616851; doi:10.1371/journal.pgph.0003941)
Supplement: S2 Text — (DOCX) [file pgph.0003941.s006.docx]

**Fake/real news task.**

**Study 1**

Fake news headlines were perceived as less accurate than the real headlines (*M*_fake_ = 1.75, *SD* = .37; *M*_real_ = 2.51, *SD* = .37, *p* < .001, 95% CI [-.78,-.72]). Among individuals who reported that they sometimes share news over social media (n=535), willingness to share fake news was lower than real news (*M*_fake_ = 0.21, *SD* = .44; *M*_real_ = 0.56, *SD* = .47; *p* < .001, 95% CI [.32,.38]). There were no differences between men and women in scores on the fake/real news task (*p* values >.75). A small but significant negative correlation was found between age and the ability to discriminate between real and fake news (*r*_(705)_= -.09, *p*=.01) and between age and accurate recognition of real news (*r*_(705)_= -.15, *p*<.001).

**Study 2**

Similar to Study 1, fake news headlines that were selected were perceived as less accurate than the real headlines (*M*_fake_ = 1.79, *SD* = .37; *M*_real_ = 2.57, *SD* = .37, *p* < .001, 95% CI[.81-.74]). Among individuals who reported that they sometimes share news over social media (n=350), willingness to share fake news was lower than real news (*M*_fake_ = 2.43, *SD* = .47; *M*_real_ = 2.80, *SD* = .46; *p* < .001, 95% CI [.33-.41]).

As in Study 1, there were no differences between men and women in scores on the fake/real news task. Replicating Study 1 findings, negative correlations were found between age and the ability to discriminate between real and fake news (*r*_(502)_=-.14, *p*=.002) and between age and accurate recognition of real news (*r*_(502)_=-.17, *p*< .001). Positive correlations between Truth-discrimination and education level (*r*_(502)_=.12, *p*< .009) and income level (*r*_(502)_=.13, *p*=.006) were found.
