# Supplementary material for: The role of epistemic trust and epistemic disruption in vaccine hesitancy, conspiracy thinking and the capacity to identify fake news
Source: PLOS Glob Public Health. 2024 Dec 4;4(12):e0003941. doi: 10.1371/journal.pgph.0003941 (PMC11616851; doi:10.1371/journal.pgph.0003941)
Supplement: S3 Text — (DOCX) [file pgph.0003941.s007.docx]

**Percentage of participants reporting on conspiracy beliefs in relation to COVID-19 conspiracy beliefs**

**Study 1**

In total, 19% (n=135) of participants in the current sample highly agreed (agreed a lot or agreed completely) with at least one of the specific COVID -19 conspiracy beliefs and 26% (n=180) highly agreed with at least one general conspiracy belief. 63% (n=480) highly agreed with at least one of the official explanations.

**Study 2**

85% (n=426) of participants in the current sample highly agreed to having the COVID-19 vaccine if offered (‘6’ or ‘5’ on a Likert scale), while 7% (n=35) disagreed to have it (‘1’ or ‘2’). In relation to confidence in the safety and efficacy of the COVID-19 vaccination programme, 72% (n=377) of participants in the current sample showed high confidence (‘6’ or ‘5’) in the programme, while 19% (n=94), showed moderate confidence (‘4’ or ‘3’) and 7% (n=31) showed low confidence (‘2’ or ‘1’).
